# Familial chylomicronemia syndrome: a case report

**DOI:** 10.1186/s13256-020-02609-0

**Published:** 2021-01-08

**Authors:** Ammu Thampi Susheela, Padmesh Vadakapet, Lekshmi Pillai, Susheela Thampi

**Affiliations:** 1 Loyola-MacNeal Hospital, 3249 S Oak Park Ave, Berwyn, IL 60402 USA; 2 Edward Hines, Jr. VA Hospital/Loyola University Medical Center, 5000 5th Ave, Hines, IL 60141 USA; 3 Ahalia Women and Children’s Hospital, P.O, Ahalia Double Rd, Ahalia Campus, Kanal Pirivu, Kerala 678556 India

**Keywords:** Chylomicronemia, Blood, Hematology, Genetic disease, Lipoprotein

## Abstract

**Background:**

Familial chylomicronemia is an extremely rare disease. Lipoprotein lipase deficiency, lipoprotein defect or lipoprotein receptor defect are the main genetic causes of familial chylomicronemia.

**Case presentation:**

We report a rare case of hypertriglyceridemia which was diagnosed at 24 days after birth. A newborn south east Asian baby born for G3P2A1 mother was presented with hematuria at 24 days at the hospital. The patient's family history is significant for pink blood in an elder sibling who died within a few months of birth without a proper diagnosis. Physical examination was not significant for any findings. Urinalysis revealed numerous RBC in the urine. Blood draw to perform renal function test revealed a characteristic pink blood. Baby’s blood was normal and red in color at the time of birth. During the present visit, although most of the blood test were not able to be carried out by the regular laboratory instruments, the patient’s lipid profile was alarmingly high with triglyceride levels over 4000 mg/dL. Due to a very high triglyceride level in a neonate and a significant familial history, a genetic cause of hypertriglyceridemia is suspected. Upon diagnosis, baby was discontinued of breast feeding completely and was given a special diet devoid of triglyceride and containing medium chain fatty acid diet and was also started with fenofibrate. After a month and a half, follow up tests were conducted which showed the triglyceride level was reduced to 1300 and a reversal of the blood color from pink to red. Since the imported diet was extremely expensive for the family, the patient was put on skimmed milk with medium-chain triglyceride (MCT) oil. With 6 weeks of treatment, baby’s condition has improved and is thriving well.

**Conclusions:**

Our case reports an extremely rare and fatal condition and illustrated the significance of timely diagnosis and intervention for saving the life of the baby.

## Introduction

In the current era, increased incidence of obesity has resulted in increased hyperlipidemia and hypertriglyceridemia [1]. However, a genetic cause of hypertriglyceridemia is rare. The mechanism of the genetic cause of hypertriglyceridemia includes lipoprotein molecular defect, lipoprotein lipase deficiency or lipoprotein receptor defect [2]. Familial hypertriglyceridemia is a fatal disease that demands early diagnosis and treatment as the high triglycerides can cause pancreatitis, lipemia retinalis, coronary heart disease, and death [2]. Here we discuss a very rare presentation of hypertriglyceridemia as hematuria and pink blood at 24 days after birth and how early diagnosis and treatment resulted in improving the condition of the baby.

## Case presentation

A 24-day old south east Asian baby was presented to the hospital with hematuria with pink urine. There were no other reported symptoms. There was no history of convulsion jaundice, fever, bleeding manifestation or skin rash. This baby is the third born to non-consanguineous parents. Since the time of birth to present, the child was fed exclusively with breastfeeding.

Family history is significant for similar symptoms in the elder sibling. The eldest sibling had seizures and pink blood. Although the child was taken to various hospitals a correct diagnosis was not made and the baby was continued on exclusive breastfeeding and died within 3 months after birth. The second born child is now 4 years old and is doing well.

Physical examination was insignificant for any findings. There were no eruptive xanthomas. The abdomen was soft and non-tender with mild hepatomegaly. There were no dysmorphic features. Vital signs at presentation on 24th day of life: heart rate 128/minute, respiratory rate 46/minute, capillary refill time (CFT) < 3 sec, SpO2 97% in room air, and temperature 36.6 deg C. A fundoscopic examination was not performed in this newborn.

**Fig. 1:**
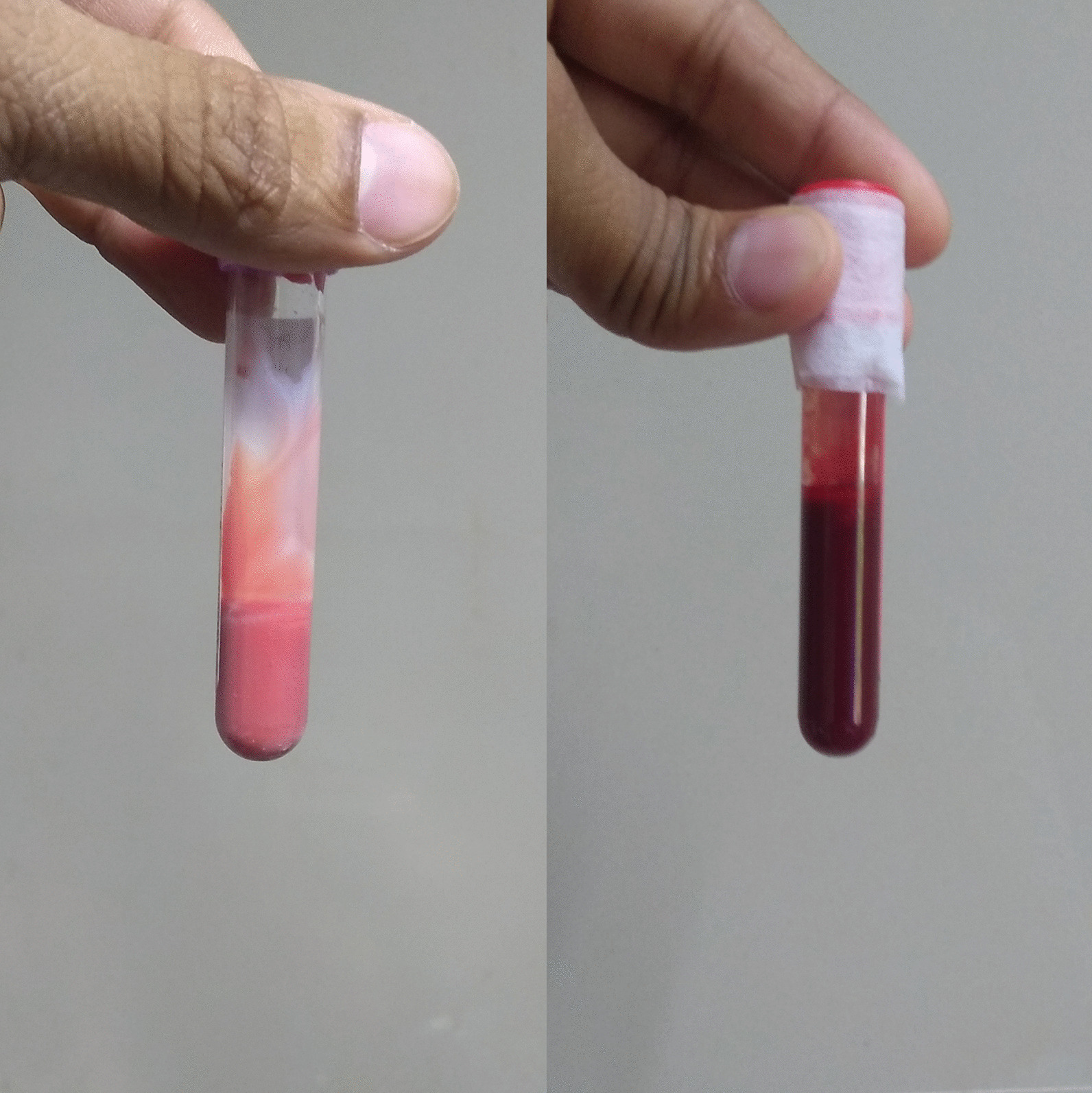
The figure shows the color change from pink to red of the blood of the neonate following the treatment of familial chylomicronemia syndrome

Urine analysis during the present visit showed pink urine with numerous red blood corpuscles. While blood draw was attempted to collect blood for renal function test, the blood was pink in color. The blood was more viscous and milkier with characteristic pink color (Figure 1). The highly milky and viscous blood was difficult to sample, and the triglyceride levels were extremely high in thousands of mg/dL. The child had a normal liver function test with sterile blood culture. Lab tests are summarized in Table 1.

**Table 1:** Lab reports

	At diagnosis	At 6 weeks	At 1 year age
Total cholesterol	Machine does not detect sample as blood	170 mg/dL	69 mg/dL
Triglycerides	> 4000 mg/dL (manually tested)	1338 mg/dL	142 mg/dL
HDL	Machine does not detect sample as blood	12 mg/dL	13 mg/dL
LDL	Machine does not detect sample as blood	38 mg/dL	28 mg/dL
VLDL	Machine does not detect sample as blood	268 mg/dL	28 mg/dL
SGOT	Machine does not detect sample as blood	43 U/L	43 U/L
SGPT	Machine does not detect sample as blood	40 U/L	24 U/L
Alkaline phosphatase	Machine does not detect sample as blood	428 U/L	287 U/L

*HDL* high density lipoproteins, *LDL* low density lipoprotein, *VLDL* very low density lipoprotein, *SGOT* serum glutamic-oxaloacetic transaminase, *SGPT* serum glutamic pyruvic transaminase

Imaging studies including ultrasound abdomen was normal. Genetic analysis was not done.

Differential diagnosis included familial hypercholesterolemia, familial triglyceredemia, and familial combined hyperlipidemia. Familial triglyceredemia was the final diagnosis of the disease due to the high level of triglycerides. Genetic testing was not done due to the limited resources and limited financial background of the family.

As a part of treatment, breast feeding was stopped, and the child was put on a special imported diet called Protein-Vitamin-Mineral Module With Iron (ProViMin) that is devoid of triglyceride. Medium chain triglycerides were additionally supplemented. The baby was also started on fenofibrate to decrease the triglyceride levels. After 3 months, repeated blood test revealed a reduction in triglyceride level to 1300 mg/dL and the color of the blood reverted from pink to red (Figure 1).

## Discussion

Hypertriglyceridemia is defined as the level of triglyceride above 95th percentiles of the corresponding age and sex. Hypertriglyceridemia can be mild to moderate (150–499 mg/dL) to severe (> 500 mg/dL). Severe hypertriglyceridemia could potentially be a fatal condition that can have primary and secondary causes. Primary hypertriglyceridemia is due to a genetic defect [1]. Secondary hypertriglyceridemia can be due to high-fat diet, obesity, diabetes, hypothyroidism, and medications such as estrogen and tamoxifen [1].

Genetic causes of hypertriglyceridemia are grouped under familial chylomicronemia syndrome [3]. Familial chylomicronemia syndrome (FCS) is a condition resulting due to the development of accumulation of chylomicrons in the plasma with one of the following manifestations such as eruptive xanthema, lipemia retinalis, and abdominal findings of pain, pancreatitis, or hepatosplenomegaly. Other manifestations of this disease include blurred vision, memory disturbances, depression, dyspnea, and flushing with alcohol intake [4]. FCS occurs in 1 in one million for homozygote and 1 in 500 for heterozygote [1]. The mechanism of familial chylomicronemia syndrome might be due to lipoprotein defect, lipoprotein lipase deficiency, or lipoprotein receptor defect [2]. Studies have reported that T108R mutation in GP1HBP1 suggests chylomicronemia [2]. 25% of cases of familial chylomicronemia syndrome manifest during infancy although extremely rare cases manifest during the neonatal period as in this case within 24 days after birth [1]. Several cases have been reported in India between 20 and 60 days of birth [1, 5, 6].

The physical presentation of the hypertriglyceridemia includes abdominal pain, eruptive xanthomas, hepatosplenomegaly, recurrent acute pancreatitis, and lipemia retinalis [1]. The heterozygous FCS can present with varying severity and usually manifest as pallor, anemia, jaundice, irritability, and diarrhea [1, 7]. A study conducted in Quebec, Canada illustrated LPL deficiency with heterozygous genotype presenting as irritability, pallor, gastrointestinal bleed, anemia, and splenomegaly [8].

Severe hypertriglyceridemia can cause pink colored blood, milky white supernatant, falsely elevated hemoglobin, and pseudohyponatremia [9]. Milky pink viscous blood is one of the characteristics of severe hypertriglyceridemia [5]. The pink color is due to the intermingling of the red blood corpuscles with the opaque white triglycerides containing very low-density lipoprotein and chylomicrons [9]. The centrifuge of the blood will yield milky white serum which is due to high levels of VLDL or chylomicrons [9]. Lab studies usually reveal very high levels of triglycerides and cholesterol. In most reported cases, hemoglobin is usually lower with high levels of triglycerides [9]. However, extreme hypertriglyceridemia can result in alteration of peripheral blood and artificially elevated hemoglobin. Automated hemoglobin measurements are based on spectrophotometric methods while other light interfering materials such as triglyceride containing particles may result in falsely elevated hemoglobin [9]. Another false test includes pseudohyponatremia as the volume of the non-aqueous phase of serum increases due to severely increased lipid levels or protein levels or radiocontrast substance or dextran. Whereas the real sodium levels remain unchanged. Pseudohyponatremia can be confirmed by using the corrected sodium formula in hypertriglyceridemia and hyperlipidemia [9]. The pink hematuria in the newborn may be due to chylohematuria as hematuria is often seen in association with chyluria [10].

Abnormal levels of triglycerides and cholesterol can lead to metabolic dysfunction and cardiac diseases [11]. Complications of FCS include pancreatitis, pancreatic necrosis, and coronary artery disease [4]. The deterioration of pancreas occurs very slowly in FCS. Pancreatic damage is due to the direct molecular effect of free fatty acids. The high concentration of free fatty acids causes reduced pH, that activates trypsinogen. Chylomicrons may damage distal pancreatic circulation that may damage pancreatic blood circulation thus inducing ischemia. The damage to the pancreatic circulation can alter the acinar function, exposing the pancreatic tissue to triglycerides. Exposure to triglycerides can activate the pancreatic lipase and induce autoinflammation [9, 12]. Chang *et al.* have identified genes responsible for hypertriglyceredemic pancreatitis [13, 14]. A serum triglyceride level greater than 203.4 mg/dL (11.3 mmol/L) indicates an increased risk of developing acute pancreatitis with incidence up to 21% [9]. Hypertriglyceridemia pancreatitis may not be diagnosed by serum amylase levels as substantial hyperamylase may not be seen in almost half of the patients [9]. The circulating inhibitor may cause suppression of enzyme activity and serum amylase [9]. However, a study done by Keim *et al.* reported that lipase is superior to amylase in detecting acute pancreatitis. Lipase at the cutoff near upper limit of normal was more predictive of acute pancreatitis after two days of abdominal pain. Simultaneous assay of lipase and amylase marginally improved the specificity of the diagnosis in patients with acute abdominal pain [15]. An ultrasound abdomen is also recommended to detect pancreatitis. Biopsy of eruptive xanthomas can be helpful for the diagnosis of hyperlipidemia.

Data for optimal treatment of familial hypertriglyceridemia and hypercholesterolemia is rare [1]. FCS due to Lipoprotein lipase (LPL) deficiency or apolipoprotein C-II (apo CII) defect is difficult to treat [1]. Previous reports have mentioned benefit from dietary restriction, lipid-lowering agent, medium chain fatty acid formula, intravenous fluid (IVF), and blood transfusion [1]. Dietary triglyceride level restriction is of paramount importance with target levels of 50 g/day or under 25% of total calorie intake to less than 20 g/day or under 15% of total calorie intake for adults [1, 16]. In neonates, breastfeeding must be stopped completely as breast milk can increase the triglyceride levels. A special diet of medium chain fatty acid oil is available [1, 2, 6].

Fibric acid derivatives such as Gemfibrozil and Fenofibrate are recommended [1]. Fenofibrate may reduce hepatic triglyceride synthesis and increase LPL activity [1]. The adverse effect of fibrates includes GI upset, cholelithiasis, elevated levels of creatinine kinase and liver enzymes [1]. Wheeler *et al.* conducted an RCT in 16 children with FCS on fenofibrate and found only 1 person developed transaminases [17]. The use of statin in infants can lead to myopathy and rhabdomyolysis [1]. Niacin is not recommended in children due to poor tolerance, serious adverse effects, and limited data [1].

Plasmapheresis may also be performed for the rapid removal of the chylomicrons [9]. Reports have shown that plasmapheresis decreases plasma triglycerides up to 70% in patients with hypertriglyceridemic pancreatitis when the standard medical therapy fails. It remains the quickest way to reduce triglycerides and early initiation can prevent end organ damage [18].

## Conclusion

Our case illustrates a rare disorder that was timely diagnosed and managed that saved the life of a newborn. Due to the rarity of the disease, there is insufficient data regarding treatment protocol for familial chylomicronemia syndrome which can present as pink blood in an infant. Hence if a patient presents with pink blood, hypertriglyceridemia must be ruled out as it is a reversible and manageable condition with dietary restrictions, special formula diet, lipid-lowering agents, and plasmapheresis.

## Data Availability

Any additional data or material is available on request.
